# Gestion des secours médicaux aux victimes des attentats terroristes au Mali

**DOI:** 10.11604/pamj.2018.30.28.14610

**Published:** 2018-05-15

**Authors:** Almeimoune Abdoulhamidou, Mangane Moustapha, Diop Madane Thierno, Beye Seydina Alioune, Démbele Seydou Aladji, Diarra Kassim, Diango Djibo Mahamane

**Affiliations:** 1 Département Anesthésie-Réanimation et Médecine d’Urgence CHU Gabriel Toure, Bamako, Mali; 2 Service Anesthésie-Réanimation et des Urgences Hôpital de Ségou, Mali; 3 Service d’Anesthésie Réanimation du CHU IOTA, Mali; 4 Service ORL CHU Gabriel Toure, Bamako, Mali

**Keywords:** Afflux massif, attentat suicide, terrorisme, CHU Gabriel Touré, urgence, Guerre, Mali, Massive influx, suicide bombing, terrorism, University Hospital Gabriel Touré, emergency, war, Mali

## Abstract

**Introduction:**

Les récents attentats suicides ont révélé un nouveau type d'afflux massif de blessés, unique en son genre, qui doivent être pris en charge au sein des formations hospitalières.

**Méthodes:**

Nous rapportons l'expérience du CHU Gabriel Toure de Bamako et de l'hôpital N. Fomba de Ségou dans la gestion des secours médicaux aux victimes des attentats terroristes au Mali.

**Résultats:**

Soixante-quatre victimes des trois attentats ont été inclus dans notre étude parmi ceci 22 (34,4%) cas de décès constaté à l'arrivée. Les hommes avaient représenté 97% des victimes qui étaient de nationalité malienne dans 71% des cas. Dans 51% des cas il s'agissait de blessé grave avec des lésions prédominantes aux extrémités chez 35% d'entre eux. Aucune victime admise vivante n était décède au cours de la prise en charge hospitalière.

**Conclusion:**

Pouvoir disposer de données précises sur les mécanismes et la gravité des lésions, une prise en charge médicale pré hospitalière permettront de diminuer la morbi-mortalité liée à ces catastrophes.

## Abstract (en)

**Introduction:**

Recent suicide bombings have revealed a new type of massive influx of injured people, unique of its kind, which must be managed in Hospitals.

**Methods:**

We here describe the experience in the management of medical assistance to the victims of terrorist attacks in Mali in the University Hospital Gabriel Toure in Bamako and in the Hospital N.Fomba in Segou.

**Results:**

Sixty-four victims of three attacks were included in our study, among them 22 (34.4%) died on arrival. Ninety-seven percent of victims were men; the victims were native of Mali in 71% of cases. Fifty-one percent of participants had severe injuries; 35% of these participants had predominant lesions on their limbs. No victim included alive died during treatment at the Hospital.

**Conclusion:**

The availability of accurate information’s on the mechanisms and the severity of the lesions as well as prehospital medical treatment will reduce disaster-related morbi-mortality.

## Introduction

Le code pénal définit l´acte terroriste comme un acte se rattachant à « *une entreprise individuelle ou collective ayant pour but de troubler gravement l´ordre public par l´intimidation ou la terreur* ». Il convient d'indiquer que si le terrorisme a toujours existé, au XXI^ème^ siècle, le nombre d'attaques, comme celui des victimes, a connu une augmentation accélérée. Ces quinze dernières années, les attentats terroristes sont passés de moins de 2.000 à près de 14.000. Quant au nombre de tués, il a été multiplié par neuf. Plus de 90% des attentats terroristes atteignent leurs objectifs immédiats, à savoir l'assassinat de civils, de policiers, de militaires ou de fonctionnaires. Ce taux de «réussite» élevé est imputable à l'utilisation généralisée d'engins explosifs artisanaux, qui sont souvent déclenchés par des kamikazes 'la pratique des attentats-suicides a aussi pris de l'ampleur. Cinquante-huit pourcent (58%) des attentats terroristes sont commis avec des bombes et 34% avec des armes à feu. Les 10% d'attaques restantes font appel à d'autres méthodes. Dans seulement 4% des cas, aussi bien des armes à feu que des explosifs sont utilisés, mais les spécialistes prévoient une augmentation de cette combinaison. En effet, cette double technique tue presque trois fois plus que le seul recours à des armes à feu [1]. Alors que le terrorisme augmente et s'internationalise, ses acteurs, ses objectifs, ses tactiques et son mode d'organisation et d'opération sont en mutation. A certains égards, les États ont renforcé leur dispositif de défense contre les terroristes, mais d'autres stratégies restent à revoir, car les pays en voie développement payent un lourd tribut. Les pays développés ne sont pas épargnés, leur vulnérabilité est réelle, comme en témoignent les attentats de Bruxelles, de Paris, de Nice courant 2016. Les répercussions de ces actes terroristes dévastateurs sont telles que certains principes ont été remis en cause, à l'image de la libre circulation ou du caractère confidentiel des communications. Le terrorisme alourdit également les dépenses publiques et rend plus difficiles les voyages, la cohabitation entre différentes communautés. Dans le contexte Malien, les groupes terroristes sont parvenus à menacer la stabilité du pays, influent ainsi sur les rapports de force géopolitiques d'où notre intérêt porter à ce sujet. L'objectif de cette étude était de partager l'expérience de l'équipe du CHU Gabriel Touré et de l'hôpital Namankoro Fomba de Ségou dans la prise en charge d'un afflux massif de blessés secondaires à des attentats.

## Méthodes

Nous avons mené une étude rétrospective exhaustive portant les victimes des trois attentats de Bamako et l'attaque du camp militaire de Nampala à 512 Km de Bamako.

**Critères inclusion:** Tous individus, blessés, ou décédés suite aux évènements sus cités, vu au service d'accueil des urgences quelques soit l'Age, le sexe, la nationalité, la profession, admission dans un centre hospitalier à Bamako et/ou à Ségou.

**Critères de non inclusions:** Victimes ou blessés balistique non liée aux attentats.

**Supports de recherche:** Registres et dossiers des blessés et victimes d'attentats au SAU CHU Gabriel Touré, dossiers et fiches d'EVASAN des blessés et victimes d'attentats de l'hôpital Namankoro Fomba de Ségou, rapport technique de la gestion des 3 attentats terroristes à Bamako fourni par la direction de la protection civile.

**Variables recueillies:** Pour chaque patient, les données suivantes ont été collectées: l'âge, le sexe, la profession, la nationalité, le mécanisme lésionnel, la localisation des lésions, le lieu de prise en charge, le type d'organisation hospitalier mise en place pour l'accueille des blessé, les principes thérapeutiques, évolution et le mode de sortie.

**Plan d'analyse:** La saisie et l'analyse des données ont été faites sur le logiciel SPSS 19.0. Le traitement de texte et les tableaux ont été réalisés avec Microsoft Word 2010. Les graphiques ont été réalisés avec Microsoft Excel 2010. Le test statistique utilisé pour la comparaison des variables était le Chi carré. Le test était significatif pour une valeur de p < 0,05 soit un intervalle de confiance de 95%.

## Résultats

**Figure 1:**
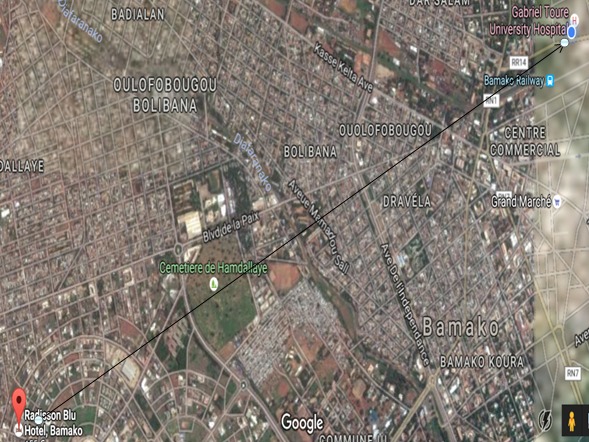
Situation géographique hôtel Radisson Blu et CHU Gabriel Toure (Google earth)

**Figure 2:**
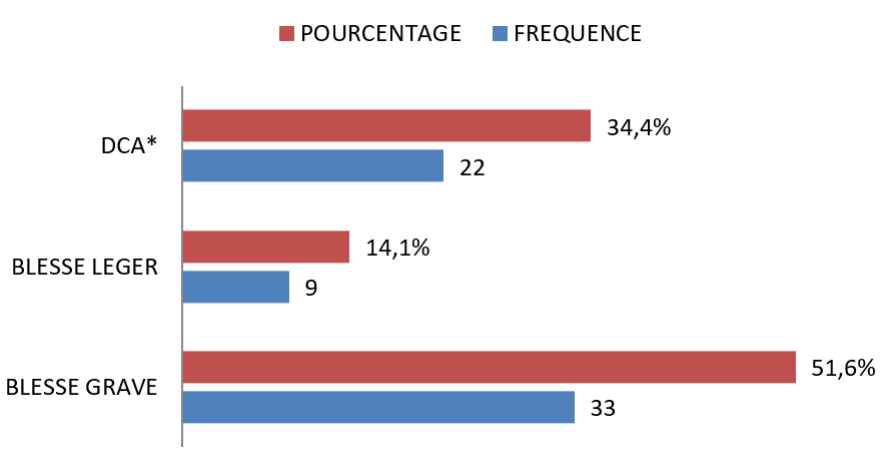
Triage, *décès constaté à l´arrivée

Soixante-quatre victimes des trois attentats ont été inclus dans notre étude parmi ceci 22 (34,4%) cas de décès constaté à l'arrivée. Le sex ratio était de 31. Aucune victime admise vivante n était décède au cours de la prise en charge hospitalière. Seul l'attentat de l'hôtel Radisson Blu a vu l'activation d'un plan catastrophe pré hospitalier sous forme d'un PMA (poste médical avancé). En absence de système de management médical pré hospitalier, dans les zones urbaines le service de la protection civile a procédé au ramassage et au transport des victimes. Ce ramassage se fait sous forme d'un «scoop and run» (Tableau 1). Ce PMA avait enregistré 14 blessés légers constituants le lot des impliqués dont prise en charge avait nécessité un soutien psychosocial. Les Moyens humains engagés pour le soutien psychosocial étaient formés par les équipes de la protection civile malienne, la croix rouge malienne, MSF-France et de la MUNISMA. Les victimes de sexe masculin avaient représenté 97% des cas, ils étaient de nationalité malienne dans 71% des cas. Dans 25 cas, soit 40% des victimes, il s'agissait de victime civile (graphique 2). Au tri d'admission 51% des cas était de blessé grave avec des lésions prédominantes aux extrémités chez 35% d'entre eux (Figure 1, Figure 2). Le caractère soudain et imprévisible de ces évènements associé à la proximité des lieux d'avec les centres hospitaliers était difficilement compatible alerte précoce d'où son absence complète lors de l'attentat de la terrasse et seulement vingt minutes avant l'admission du premier blessé lors de l'attaque de Radisson Blu (Tableau 2). L'état de 37 blessés était compatible avec une hospitalisation standard en service de chirurgie et en traumatologie, 3 cas ont fait l'objet d'un déchoquage préalable puis admission au bloc opération pour respectivement hémostase, chirurgie digestive et extraction de corps étrangers (Tableau 3). Les suites opératoires se sont émaillées d'une péritonite post opératoire. Les vingt patients sur vingt-deux décédé avant l'arrivée à l'hôpital portaient tous des lésions de multicriblage balistique.

**Tableau 1:** Récapitulatif des plans catastrophe des attentats au Mali

Attentats	Lieu	Plan catastrophe pré hospitalière	Ramassage et transports	Plan catastrophe hospitalière
Terrasse	Bamako	Non	Protection civile	Non
Radisson Blu	Bamako	PMA^+^	Protection civile	Plan blanc CHU G. Toure
Nampala	Ségou	Non	Armé et particuliers	Plan blanc hopital Namankoro Fomba

**Tableau 2:** Délai entre alerte hospitalier et l’admission du premier blessé

ATTENTATS	CHU GABRIEL TOURE	HOPITAL N. FOMBA/ SEGOU
TERRASSE	PAS D’ALERTE	-
RADISSON BLU	20 MIN	-
NAMPALA	-	60 MIN

**Tableau 3:** Orientation au triage

ORIENTATION	FREQUENCE	POURCENTAGE
DECHO+BLOC OP+REA	3	4,7
CHIRURGIE	8	12,5
TRAUMATO	14	21,9
EVASAN* Bamako	2	3,2
BOX (observation)	13	20,3
MORGUE	22	34,4
AUTRES	1	1,6
TOTAL	64	100,0

* Evacuation sanitaire

## Discussion

**Figure 3:**
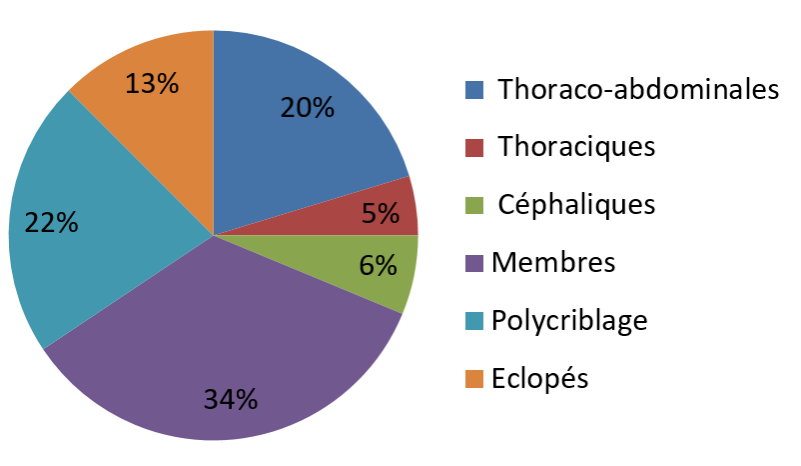
Répartition des lésions anatomiques

**Ramassage et caractérisation des afflux massifs post attentats:** Les conflits récents ont amené une transformation des zones de combat en guérilla urbaine aboutissant à une désorganisation des principes de ramassage et à un engorgement des structures hospitalières d'accueil, cette dernière est connue sous le terme « main gate syndrom » [2]. Le caractère imprévisible de l'horaire et le lieu de la détonation des attentats, la capacité des agresseurs à se fondre dans la masse et la proximité des explosions, des endroits les plus passants des grandes villes ramènent le « point zéro » au plus proche des portes d'entrées « Main Gate » de la structure hospitalière. Une des premières conséquences est de diminuer le délai d'arrivée des premiers blessés dans la structure d'accueil. Ce caractère a été retrouvé au cours des attentats au mali dans trois cas comme illustré par la Figure 3.

**Nombre de blessé et triage:** Dans notre expérience la première victime était admise au SAU du CHU Gabriel Touré 20 min après l'alerte soit près 60 min après impact. Des travaux récents [3] estiment que 34% des blessés arrivent dans un délai de 10 min et 65 % des blessés sont pris en charge dans les 30 min après l'heure zéro de l'explosion. Mais du fait de cette proximité, le ramassage classique avec catégorisation et une mise en condition avant évacuation était inefficace. Aucune de nos victimes, même les plus graves n'avaient une voie veineuse périphérique à leurs admissions aux urgences. Ce système de ramassage permet un délai de transport diminué par rapport aux situations classiques ainsi les blessés qui traditionnellement étaient mourants ou considérés en urgence dépassée peuvent bénéficier d'une chirurgie de ressuscitation immédiate. En illustration, nous rapportons le cas d'une victime de l'attentat de hôtel Radisson blu, il s'agissait d'un patient multi- criblé balistique thoraco-abdominale avec hémo-pneumothorax suffocant et état de choc. Le drainage thoracique avait ramené d'emblée 1000 ml de sang indiquant une thoracotomie d'hémostase. Ce dernier geste a été salvateur. Cependant l'incertitude sur l'arrivée d'autres victimes, la possibilité que des connaissances du personnel hospitalier soient au nombre des victimes et le risque d'une seconde explosion intensifient l'atmosphère chaotique qui existe déjà, en règle générale, aux urgences. Dans notre expérience nous n'avons pas enregistrés un nombre significatif d'éclopé civil à Bamako, à Ségou on notait 5 éclopés militaires, contrairement aux équipes irakiennes [4], où les blessés les plus graves ne sont pas nécessairement les premiers à arriver au triage les éclopés arrivant en premier par leurs propres moyens, sans mise en condition préalable, augmentant la charge de travail du personnel des urgences. Dans notre contexte le triage est rendu difficile par l'absence de médecine pré hospitalière. Le ramassage et le transport des victimes se résument à un système de “scoop and run”' par les sapeurs-pompiers dans des conditions logistiques précaires voire inexistant. L'engorgement du SAU était lié à la difficulté de transfert intra-hospitalier des patients déjà présent dans la structure.

**Mécanisme lésionnel:** Tous les attentats que nous rapportons, ont été perpétrés par des armes à feu sous le mode de fusillade, du coup la quasi-totalité des lésions étaient de type balistique. Ces faits qui contrastent avec les conflits Irakiens où le mécanisme lésionnel le plus fréquemment rencontré était les explosions dans 73% des cas [4].

**Localisations des lésions:** Les blessures des extrémités et les lésions thoraco-abdominales étaient dominantes dans le contexte malien comparativement à leur répartition chez les militaires de l'OTAN en Irak ou la majorité des lésions étaient localisés au niveau céphalique et aux extrémités. La raison de cette différences de répartition des lésions pourrait être due aux faits que les militaires sont protégés par les gilets pare-balle thoraco-abdominale contrairement aux populations civiles. Quant aux victimes maliennes presque, la moitié (49,4%) était des civiles et des paramilitaires sans protections pare-balle (Tableau 4).

**Tableau 4:** Llésions traumatiques et auteurs

LESIONS ANATOMIQUES	MALI	IRAK [4]
ABDOMEN-THORAX-PELVIS	13 (20,7%)	709 (10,6%)
THORAX	3 (4,7%)	376 (5,6%)
CEPHALIQUE	4 (6,3%)	1949 (29,4%)
MEMBRES	22 (35,1%)	3573(54,1%)
NON RENSEIGNER	14 (21,9)	-
PAS DE LESIONS	8 (12,6)	-

**Gravité des lésions:** La gravité des blessures de guerre à augmenter au cours des conflits actuel. Le mécanisme lésionnel lui-même est à l'origine de blessure de gravité croissante.

**Cause des décès:** Nous n'avons enregistré aucun décès hospitalier ce qui dénote d'une certaine célérité dans la prise en charge hospitalière. Malgré cela 22 cas de décès ont été constaté à l'arrivée au SAU du CHU Gabriel Touré ce qui pose un certain nombre de question: Ces décès étaient-ils évitable? Quelle était le délai entre l'impact et prise en charge? Ballamy et al, au Vietnam [5] met en évidence que 70% des soldats décédés meurent dans les cinq premières minutes de leur blessure, tandis que 20 % meurent encore avant de recevoir les premiers soins médicaux. Actuellement en Irak, ce délai d'évacuation reste cependant rapide avec une moyenne de 60 minutes entre la blessure et l'accès à une formation sanitaire de rôle 2 [6]. Quelles étaient conditions de ramassage et de management pré hospitalier? Etaient-ils en état choc hémorragique? Ou asphyxiés par pneumothorax suffocant?, ces questions ont été déjà évoquées par Sauaia et al [7] pour qui, il existe des similitudes entre les blessés des conflits et la traumatologie civile: où les blessés meurent essentiellement dans les premières heures qui suivent l?accident, de choc hémorragique ou des conséquences d'un traumatisme crânien grave. Ou alors étaient-ils en urgence dépassée? Ces questions sont difficile à répondre cependant le paramètre le plus incriminé semble être le délai entre l'heure d'impact et la nature des lésions. Le cas de l'attaque du camp de Nampala était édifiant. L'assaut a été donné à 05 heures à l'aube et le premier blessé était admis au service des urgences de l'hôpital N. Fomba de Ségou à 10h 19min, soit un délai de 5 heures.

## Conclusion

Les actes d'attentat terrorisme se multiplient dans les grands centres urbains partout dans le monde ramenant les pathologies de guerre aux portes des hôpitaux civils. Le flot de victime décédée avant admission nous interpelle sur la qualité notre système de ramassage.

### Etat des connaissances actuelles sur le sujet

- Les actes d'attentat terroriste prolifèrent dans les centres urbains sur un mode d'EEI ou suicide par ceinture explosive;
- Des vagues de victime (psychologique et somatique) débarquent dans les hôpitaux engendrant une situation d'afflux massif aux services d'accueil des urgences.

### Contribution de notre étude à la connaissance

- Au Mali les actes d'attentats terroristes ont été perpétrés par des fusils d'assaut;
- Seuls les blessés somatiques sont arrivés au S.A.U, les éclopés et les petits blessés auto soignés se sont dissipés dans la nature du coup l'afflux massif décrit n'a jamais eu lieu.

## Conflits d’intérêts

Les auteurs déclarent ne déclarent aucun conflit d'intérêts.
